# Expression of MUC16/CA125 Is Associated with Impaired Survival in Patients with Surgically Resected Cholangiocarcinoma

**DOI:** 10.3390/cancers14194703

**Published:** 2022-09-27

**Authors:** Maximilian N. Kinzler, Falko Schulze, Steffen Gretser, Nada Abedin, Jörg Trojan, Stefan Zeuzem, Andreas A. Schnitzbauer, Dirk Walter, Peter J. Wild, Katrin Bankov

**Affiliations:** 1 Department of Internal Medicine I, University Hospital Frankfurt, Goethe University, 60590 Frankfurt am Main, Germany; 2 Dr. Senckenberg Institute of Pathology, University Hospital Frankfurt, Goethe University, 60590 Frankfurt am Main, Germany; 3 Department of General, Visceral, Transplant and Thoracic Surgery, University Hospital Frankfurt, Goethe University, 60590 Frankfurt am Main, Germany; 4 Frankfurt Institute for Advanced Studies (FIAS), 60438 Frankfurt am Main, Germany; 5 Frankfurt Cancer Institute (FCI), University Hospital Frankfurt, Goethe University, 60590 Frankfurt am Main, Germany

**Keywords:** cholangiocarcinoma, mucins, CA-125 antigen, survival, surgical oncology

## Abstract

**Simple Summary:**

MUC16/CA125, a commonly used blood biomarker of ovarian cancer, is associated with cancer proliferation in several tumor entities. The data on MUC16 expression in cholangiocarcinoma (CCA) tissue are very limited. We observed a remarkable proportion of MUC16 (+) patients with surgically resected CCA as 57 of 168 (34%) patients with CCA had evidence of MUC16 expression in tumor tissue. We found a significantly impaired overall survival for MUC16 (+) patients (27.4 months) in comparison to MUC16 (−) patients (56.1 months). Our data demonstrate that MUC16 (+) is an independent risk factor for poor survival in CCA patients.

**Abstract:**

MUC16/CA125 is associated with cancer proliferation in several tumor entities. The data on MUC16 expression in cholangiocarcinoma (CCA) tissue are very limited. The aim of this study was to assess the MUC16 status and its impact on survival in CCA patients. All the patients with surgically resected CCA that were diagnosed between August 2005 and December 2021 at the University Hospital Frankfurt were retrospectively analyzed. A 7-Mucin biomarker panel was assessed by immunohistochemistry. For overall survival (OS), Kaplan–Meier curves and Cox-regression analyses were performed. Randomly selected intrahepatic cholangiocarcinoma (iCCA) were further processed for differential expression profiling. A total of 168 patients with CCA were classified as MUC16 (−) (66%, n = 111) and MUC16 (+) (34%, n = 57). Subgroup analyses revealed a median OS of 56.1 months (95% CI = 42.4–69.9 months) and 27.4 months (95% CI = 15.8–39.1 months) for MUC16 (−) and MUC16 (+), respectively (*p* < 0.001). In multivariate analysis, MUC16 (+) (HR = 1.6, 95% CI = 1–2.6, *p* = 0.032) was an independent risk factor for poor prognosis. Prominently deregulated pathways have been identified following MUC16 expression, overrepresented in cell cycle and immune system exhaustion processes. These findings suggest including MUC16 in clinical routine diagnostics as well as studying its molecular pathways to identify further mechanistic key players.

## 1. Introduction

Cholangiocarcinoma (CCA) represents a heterogenous group of highly malignant cancers with poor prognosis. Despite relatively rare occurrence, CCA is the second most common primary hepatic cancer after hepatocellular carcinoma (HCC) and its incidence is increasing globally [1,2]. Depending on its localization, CCA can be divided into intrahepatic (iCCA), perihilar (pCCA), or distal (dCCA) harboring distinct clinical and pathological features. The only curative therapy for all subclasses is surgical resection (R0 resection). However, even for CCA that is resected with curative intent, the prognosis is poor as most patients are not cured: the median survival ranges from about 30 months for iCCA to 38 months for pCCA [3]. Due to this devastating outcome of patients with CCA, the identification of further potential prognostically relevant markers is of utmost importance to improve the clinical management of these patients.

Mucins are high molecular weight glycoproteins that are produced by epithelial cells that serve multiple functions including lubrication, cell signaling, and maintaining epithelial integrity by forming a chemical barrier [4]. However, the aberrant expression of mucins has been reported to promote cancer development in several entities including CCA [5,6,7,8,9] by mimicking epithelial composition and structure, thus evading potential immune surveillance by growth factor and cytokine capture motifs.

MUC16 expresses the peptide epitope cancer antigen 125 (CA125), a routinely used blood biomarker of ovarian cancer, promoting cancer cell proliferation while inhibiting the anticancer immune response [10]. Furthermore, a crucial role of MUC16 is also suggested in pancreatic, colorectal, and non-small cell lung cancer [11,12,13] while its overexpression in breast cancer tissue regulates proliferation and anti-apoptosis via JAK-STAT signaling [6].

Apart from few studies evaluating the impact of preoperative CA125 serum levels on outcome [14,15], studies investigating the role of MUC16 expression in tumor tissue from CCA patients remain scarce so far. In more detail, only two studies indicated that the expression of MUC16, as well as the co-expression with mesothelin, is a risk factor for poor outcome in mass-forming iCCA and pCCA, respectively [16,17].

As the outcome of CCA patients remains poor even after surgical resection, novel therapeutic strategies for CCA patients are of high interest. Intriguingly, MUC16 has emerged as a potential target for novel cancer therapies using monoclonal antibodies or immunotherapy, particularly for ovarian and pancreatic cancer [18,19,20].

To test this hypothesis, we assessed the frequency of MUC16 in a large retrospective cohort of CCA patients that were treated in our tertiary hospital. In conclusion, this study evaluates the impact of MUC16/CA125 expression and potential pathway alterations in patients with CCA on survival after resection in curative intent for all known histopathological subtypes in a large cohort and its potential role as a prognostic marker.

## 2. Materials and Methods

### 2.1. Database and Study Population

All patients that were treated with surgically resected (R0, R1) cholangiocarcinoma at Frankfurt University Hospital between August 2005 and December 2021 were retrospectively analyzed. Histopathological confirmation was assessed independently by expert pathologists of the Dr. Senckenberg Institute of Pathology, University Hospital Frankfurt. Clinical data (date of birth, gender, tumor stage, tumor size, comorbidities, laboratory parameters, and follow-up) were collected from electronic medical records. The tissue samples that were used in this study were provided by the University Cancer Center Frankfurt (UCT). Written informed consent was obtained from all the patients and the study was approved by the Institutional Review Boards of the UCT and the Ethical Committee at the University Hospital Frankfurt (project-number: SGI-13-2018).

### 2.2. Tissue Microarray (TMA) Construction and Immunohistochemistry (IHC) Analysis

**Figure 1:**
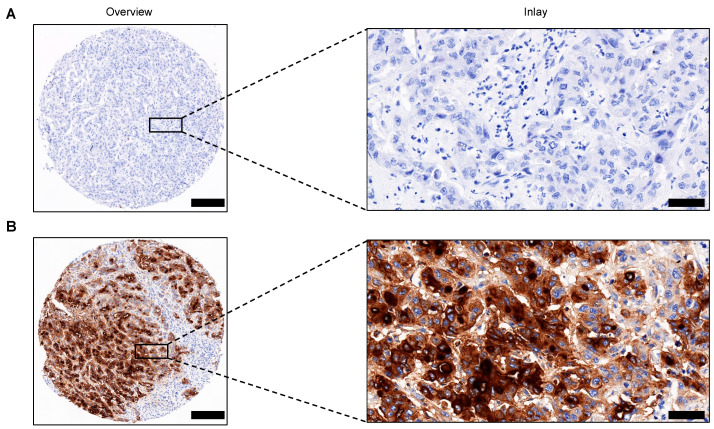
Representative images of MUC16 expression in CCA. (**A**,**B**) Representative immunohistochemistry of the absence (**A**) and presence (**B**) of MUC16 expression in TMA cores of CCA patients. Original magnification ×8.5 for overview and ×40 for inlay, respectively. Scale bars: 200 µm for overview and 50 µm for inlay, respectively. Abbreviations: Tissue Microarray (TMA).

Formalin-fixed paraffin-embedded (FFPE) tissue samples were retrieved from the archive of the Dr. Senckenberg Institute of Pathology, University Hospital Frankfurt. For the construction of TMA, annotations of defined regions of interest (ROI) containing representative tumor area were set to a core diameter of 1 mm. Via slide overlay function, digital annotations were adapted to the donor tissue and transferred to a blank recipient paraffin matrix using the TMA Grandmaster (3DHISTECH, Budapest, Hungary). HE-stained slides were automatically processed on a Tissue-Tek Prisma Plus staining device (Sakura Finetek, Torrance, CA, USA). IHC was conducted using the DAKO FLEX-Envision Kit (Dako/Agilent, Santa Clara, CA, USA) and the fully automated DAKO Omnis staining system (Dako/Agilent, Santa Clara, CA, USA) according to the manufacturer’s instructions. We performed staining of MUC1 (Clone: E29; ready to use; incubation time: 15 min; Dako/Agilent, Santa Clara, CA, USA), MUC2 (Clone: CCP58; ready to use; incubation time: 30 min; Dako/Agilent, Santa Clara, CA, USA), MUC4 (Clone: 8G7; dilution: 1:200; incubation time: 30 min; Bio SB, Santa Barbara, CA, USA), MUC5AC (Clone: CLH2; ready to use; incubation time: 30 min; Dako/Agilent, Santa Clara, CA, USA), MUC6 (Clone: MRQ20; dilution: 1:500; incubation time: 30 min; Cell Marque, Rocklin, CA, USA), MUC16 (Clone: M11; ready to use; incubation time: 60 min; Dako/Agilent, Santa Clara, CA, USA), Ki-67 (Clone: MIB-1 ; ready to use; incubation time: 20 min; Dako/Agilent, Santa Clara, CA, USA), and PD-L1 (Clone: 22C3 ; ready to use; incubation time: 40 min; Dako/Agilent, Santa Clara, CA, USA). The stained slides were scanned with the Pannoramic slide scanner (3DHISTECH, Budapest, Hungary). For MUC antibodies, staining in more than 1% of the tumor cells was considered positive. IHC analysis was performed by two independent investigators. The assessment of Ki-67 and PD-L1 was performed by a pathologist with three years of experience (S.G.). TMA cores with either the absence of representative tumor tissue or the presence of staining artifacts were excluded from the analysis. Representative images of absence and presence of immunohistochemical expression of MUC16 in CCA tissue are shown in Figure 1. A representative control image of MUC16 staining in cancerous and paracancerous tissue is shown in Supplementary Figure S3.

### 2.3. Ribonucleic Acid (RNA) Isolation and Immune Exhaustion Expression Analysis

Representative tumor material of the primary tumor was retrieved by a 1 mm core. The RNA was isolated using the truXTRAC FFPE total NA Kit (Covaris, Woburn, MA, USA) based on focused ultrasonification and column purification according to the manufacturer’s instructions. Nanostring nCounter^®^ Platform and Immune Exhaustion Panel v1 were used to enrich a commercially available function-specific panel of 798 genes by hybrid capture technique (Nanostring, Seattle, WA, USA). Nanostring nSolver™ software v4 and implemented nCounter^®^ Advanced Analysis module v2.0.115 were used for subsequent raw data processing and normalization by internal controls following differential supervised analysis between MUC1-positive (n = 7) and -negative CCA patients indicated by previous immunohistochemistry. ClueGO v.2.5.6 and CluePedia v.1.5.6 functional classification and network annotation was applied in order to identify enriched genes that were associated to overrepresented gene ontologies based on REACTOME_pathways (updated 17.08.2022) [21,22]. The Ingenuity Pathway Analysis (IPA v.76765844; QIAGEN, Hilden, Germany) tool was additionally used to predict network-associated biomarkers as well as functional terms (Fx) and therapeutical agents (Rx).

### 2.4. Statistical Analysis

We compared baseline clinicopathological characteristics between patients with an absence and presence of MUC16. The categorial variables are presented as frequencies and percentages and the continuous variables are shown as means with standard deviations. The categorial and continuous variables were compared using the Student’s *t*-test and chi-square test, respectively. The overall survival (OS) was defined as the time of onset of disease until death or date of last follow-up. The date of last follow-up was treated as censored observation.

Survival was compared using the log-rank test. The Kaplan–Meier curves for survival were derived to visualize the comparison between the absence and presence of MUC16 expression. Cox regression analysis was performed to assess the risk factors influencing patient survival. We preliminarily used univariate Cox regression analysis to screen our variables. We then included the variables with *p* < 0.05 into the multivariate Cox regression analysis. The adjusted common odds ratios are reported with 95% CIs to indicate statistical precision. The significance level was set to *p* < 0.05.

For disease survival-rate, patients without recurrence and patients who were alive at the time of data collection were counted as patients with disease survival as we only enrolled patients with surgically resected CCA with curative intent. Furthermore, patients that were treated as censored observation due to lost to follow-up were separately classified as “lost to follow-up” in this analysis. Patients for whom the exact cause of death could not be determined were classified as “not available”. We used a strict definition for cancer-related death as only patients who died in a palliative setting in our hospital, patients with dedicated palliative care treatment, patients in best-supportive care situation as well as patients who died from CCA therapy (e.g., side effects from chemotherapy) were defined as cancer-related death. Furthermore, patients with postoperative death (during the same hospital stay as surgery) were classified as cancer-related death as well. All the data were analyzed with SPSS 27 (IBM; Armonk, NY, USA) statistical software.

## 3. Results

### 3.1. Patients and Clinical Characteristics

**Figure 2:**
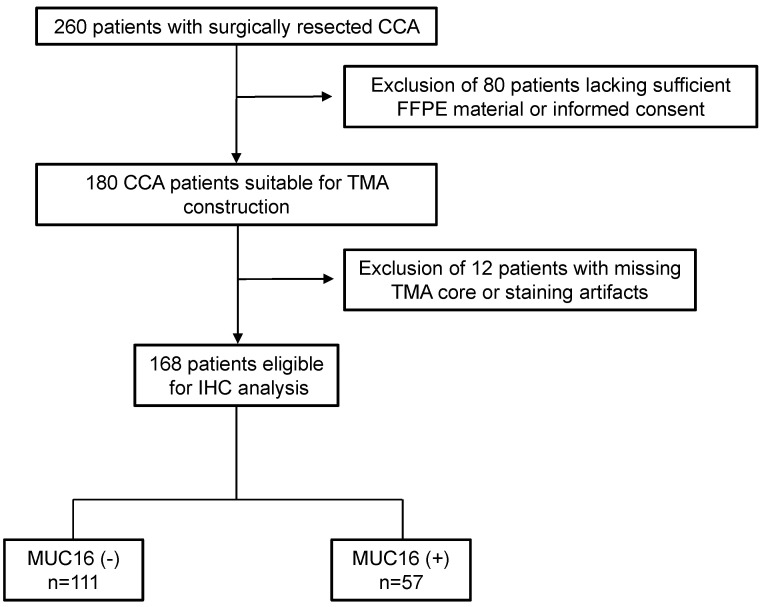
Workflow for screening, enrolment, and allocation. Abbreviations: cholangiocarcinoma (CCA), immunohistochemistry (IHC), tissue microarray (TMA).

In total, 260 patients with surgically resected CCA in our tertiary hospital were analyzed in this study. A total of 69.2% (n = 180) of the patients were suitable for TMA construction. Of the remaining 168, 66% (n = 111) were classified as MUC16 (−) while 34% (n = 57) were MUC16 (+) (Figure 2). The MUC16 (+) patients had more frequently positive serum CA-19/9 (*p* = 0.011), higher Pn status (*p* = 0.034), and increased Ki-67 proliferative index (*p* = 0.027). In contrast, MUC16 (−) was associated with larger tumor size (*p* < 0.001). Remarkably, MUC16 was more frequently expressed in iCCA compared to extrahepatic CCA (*p* < 0.001). MUC16 (−)/(+) patients with CCA did not differ in any other clinicopathological findings including common risk factors such as viral hepatitis, primary sclerosing cholangitis, liver cirrhosis, or cholelithiasis. As expected, the serum levels of CA125 were determined in only a very small minority during clinical routine diagnostics (n = 8). The baseline clinicopathological characteristics are summarized in Table 1. The details on distinct patterns of recurrence are depicted in Supplementary Table S4.

### 3.2. Impact of MUC16 Expression on Overall Survival

**Figure 3:**
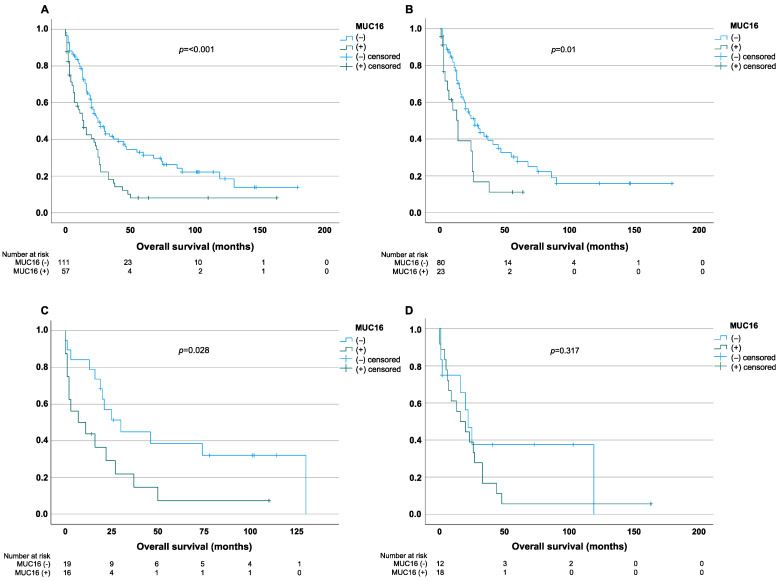
Kaplan–Meier curves for the overall survival in MUC16 (−)/(+) patients with CCA. (**A**–**D**) The overall survival that was assessed for immunohistochemical expression of MUC16 for all types of CCA (**A**), iCCA (**B**), pCCA (**C**), and dCCA (**D**). The date of last follow-up was treated as a censored observation. Abbreviations: cholangiocarcinoma (CCA), intrahepatic cholangiocarcinoma (iCCA), distal cholangiocarcinoma (dCCA), perihilar cholangiocarcinoma (pCCA).

The median OS for all 180 patients with surgically resected CCA that were included in this study was 29.1 months. In line with the literature, the Kaplan–Meier curves revealed a significant impact on OS in CCA patients for the expression of MUC4 (*p* < 0.001) or MUC5AC (*p* = 0.006) while there was no difference in the survival for MUC1 (*p* = 0.44), MUC2 (*p* = 0.3) and MUC6 (*p* = 0.983) in our study cohort [23]. In addition, the Kaplan–Meier curves indicated a median OS of 56.1 months (95% CI = 42.4–69.9 months) for MUC16 (−) in comparison to 27.4 months (95% CI = 15.8–39.1 months) for patients with MUC16 (+), thus showing a significant difference between both groups (*p* < 0.001) (Figure 3A). To further investigate the impact of MUC16 expression on OS, the three CCA subtypes were analyzed separately. In iCCA, the OS rates were 53.4 months (95% CI = 37–69.9) for MUC16 (−) patients and 19.1 (95% CI = 10.6–27.6) for MUC16 (+) (*p* = 0.01) (Figure 3B). In line with these findings, for MUC16 (−)/(+) pCCA patients, the median OS was 57.8 months (95% CI = 32.2–83.4) and 20.8 (95% CI = 5.9–35.7), respectively (*p* = 0.028) (Figure 3C). Correspondingly, MUC16 (+) is associated with impaired OS rates in patients with dCCA (26.6 months (95% CI = 10–43.2)) in contrast to MUC16 (−) patients (52.7 (95% CI = 19.9–85.4)) although these results did not reach statistical significance (*p* = 0.317) (Figure 3D). Additionally, the disease survival rate was higher in MUC16 (−) compared to MUC16 (+) patients. However, these results did not reach statistical significance (*p* = 0.385) (Table 1).

### 3.3. Risk Factors Correlating with OS in CCA Patients

As our results indicate a marked impact of MUC16 expression on survival rates in our study, we further performed univariate and multivariate Cox regression analysis to identify the correlating risk factors. Interestingly, the univariate analysis determined MUC16 (+) as a significant risk factor of OS (HR = 1.9, 95% CI = 1.3–2.8, *p* < 0.001). In addition, the presence of tumor marker CA-19/9 could be described as a significant risk factor as well (HR = 2.1, 95% CI = 1.4–3.2, *p* < 0.001). Furthermore, multiple tumors (HR = 1.8, 95% CI = 1.2–2.6, *p* = 0.002), pathological grade 3 (HR = 4.8, 95% CI = 1.2–20, *p* = 0.031), ECOG 1 (HR = 2.7, 95% CI = 1.8–3.9, *p* < 0.001), M1 status (HR = 2.7, 95% CI = 1.5–4.9, *p* = 0.001), and R1 status (HR = 1.6, 95% CI = 1.1–2.4, *p* = 0.018) also served as significant risk factors in univariate analysis. To further investigate independent risk factors, multivariate analysis was performed. Multivariate Cox regression analysis revealed that MUC16 (+) (HR = 1.6, 95% CI = 1–2.6, *p* = 0.032), ECOG 1 (HR = 2, 95% CI = 1.3–3.2, *p* = 0.003), multiple tumors (HR = 1.7, 95% CI = 1.1–2.7, *p* = 0.025), as well as the presence of tumor marker CA-19/9 (HR = 1.6, 95% CI = 1–2.6, *p* = 0.035) serve as independent risk factors for overall survival for CCA patients in our study (Table 2).

### 3.4. MUC16 Expressing iCCA Display a Differential Expression Profile

**Figure 4:**
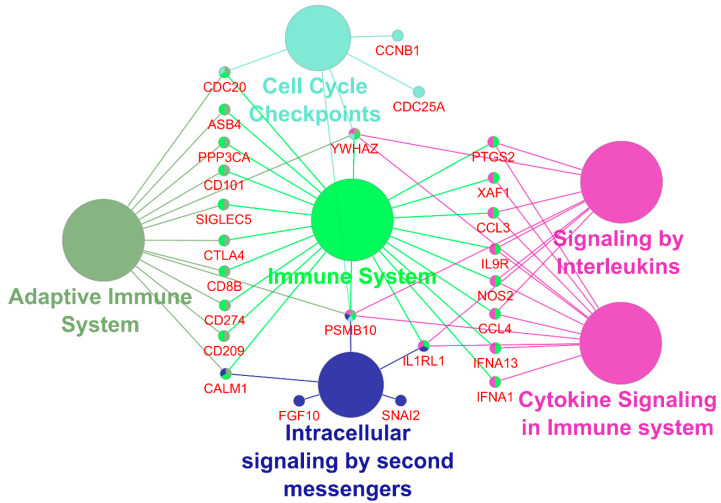
Top Genes from all the clusters that are associated with five representative terms and pathways using REACTOME_pathways ontology database. A total of 33 of top 40 prominently deregulated genes are statistically overrepresented in a functional network of ‘Intracellular signaling by second messengers’, ‘Signaling by Interleukins’ and ‘Cytokine Signaling in Immune System’, ‘Cell Cycle Checkpoints’, ‘Adaptive Immune System’ and ‘Immune System’ (Term *p*-value and group *p*-value corrected by Bonferroni step down below 0.01, Final Kappa Score groups = 4, redundant groups merged with >50.0% overlap.

Randomly selected iCCA have been included for preliminary expression analysis in dependence of MUC16 status. In total, 324 genes have been identified as differentially regulated among groups with a log2 fold change that was larger than 1 (linear fold change ≥2) and a *p*-value ≤ 0.05. A *p*-value correction that was based on the Benjamini–Yekuteli method (BY *p*-value) allowed 197 genes to be selected as prominently deregulated between groups (BY *p*-value ≤ 0.2). The top 40 candidates (BY *p*-value ≤ 0.04, log2 fold change ranging from 2–4.71) have been selected for further functional classification and are displayed in a volcano plot (Supplementary Figure S1, Supplementary Table S1). The selection revealed distinct pathways such as cell cycle checkpoint processes (e.g., CCNB1 among others); cytokine and interleukin signaling in immune system (e.g., CCL3, CCL4 among others); and pathways of the adaptive immune system highlighting checkpoint key players such as CTLA4, CD274 (PD-L1), and intracellular signaling by second messengers (e.g., SNAI2, FGF10 among other) that may be altered, providing a first tentative insight into the molecular mechanisms in MUC16-expressing tumors (Figure 4, Supplementary Table S2). Next, we performed verification of PD-L1 as a clinically relevant target by IHC in the corresponding patient tissue. In line with our expression data, IHC assessment revealed 5.9% and 75% PD-L1-positive CCA tissue in MUC16 (−) or MUC16 (+) patients, respectively. Thus, positive PD-L1 status differed significantly between MUC16 (−)/(+) tissue (*p* < 0.001). Ingenuity pathway analysis (IPA) was further used to merge networks that were associated with cancer growth and survival to predict the activation and inhibition of additional biomarkers (Supplementary Figure S2). Besides the experimentally identified biomarkers Stat3, MYC, CXCL5, CCND1, and RELA among others are predicted to be activated. The given candidates are involved in cancer cell proliferation or resistance to chemotherapy. Additional significant expression changes are depicted in Supplementary Table S3.

## 4. Discussion

The overexpression of MUC16 has been linked to worse prognosis in several malignancies [16,24,25]. Immunohistochemical expression of MUC16 in CCA has been studied to a limited extend, while novel prognostically relevant markers for this devastating tumor entity are urgently needed. Hence, the aim of the present study was to determine the impact of MUC16 expression on OS of patients with CCA after surgical resection. To our knowledge, this study is the first to investigate this clinically relevant issue in all CCA subtypes by immunostaining and differential expression analysis in order to preliminarily elucidate the pathomechanism behind its expression.

In the present study, 66% of the patients were MUC16 (−), while 34% expressed MUC16. We thereby observed a lower proportion of MUC16 (+) patients compared to data from Higashi et al., although these data solely refer to mass-forming iCCA [16]. Besides the prevalence of MUC16 expression, we also analyzed its impact on clinical outcome. We found a significantly impaired overall survival for MUC (+) patients (27.4 months) in comparison to patients without MUC16 expression (56.1 months). Several aspects need to be discussed as potential cofactors for the negative impact of MUC16 (+) on survival, i.e., MUC16 (+) patients had higher Pn status, more frequently positive serum levels of tumor marker CA-19/9, and increased Ki-67 proliferative index. Especially, R-status, metastatic spread, recurrence, and common risk factors for CCA were comparable between both subgroups. In multivariate analysis, MUC16 (+) remained an independent risk factor for impaired survival as well as the known prognostic factor ECOG 1, multiple tumors, and the presence of tumor marker CA-19/9.

In general, MUCs and their impact on survival are extensively studied in biliary tract cancer (BTC). Among these, MUC4 as well as MUC5AC are highly tumor-associated in BTC [26]. In the literature, the immunohistochemical detection of MUC1 and MUC4 serve as predictors of poor outcome in CCA [23,27] whereas we demonstrated a significant impact on survival only for MUC4 but not for MUC1 or MUC2. However, the impact of MUC1 on survival in the study from Park et al. was based solely on univariate analysis [23]. In addition, cellular and serum MUC5AC were thought to be related to advanced tumors and poor prognosis, respectively [23,28]. In contrast, a recently published study reported that the low expression of MUC5AC and MUC6 predicts poor prognosis solely for pCCA patients [29] whereas our study revealed a significant impact on OS for high MUC5AC expression. Additionally, the immunohistochemical expression of MUC6 is thought to be associated with well-differentiated CCA but not with poor survival, in line with our data [23]. However, data on the impact of MUC16 expression on the outcome of CCA patients are very limited as two studies only investigated the role of MUC16 in mass-forming iCCA or its co-expression with mesothelin in extrahepatic CCA, preventing comparability between both cohorts [16,17]. For the first time, we provide conclusive data on MUC16 expression in all three CCA subtypes from one cohort of our tertiary hospital, strengthening the role of MUC16 as a prognostically relevant marker. However, it still remains elusive whether MUC16 detection in tumor tissue and serum levels of CCA patients correlate or have similar prognostic value. In CCA patients with available CA125 serum levels, 100% (4/4) of MUC16 (−) patients were also negative for serum CA125 while 75% (3/4) of the MUC16 (+) patients were also positive for serum CA125 in our study, which may serve as the first indication of a possible correlation between MUC16 assessment by IHC and serum levels. However, these data have to be analyzed carefully as the patient cohort with available CA125 serum levels is very small in our study. As pre-operative serum levels of CA125 were recently found to predict poor prognosis in pCCA receiving surgery [14], we hypothesize that CA125 may play a crucial role in the future, especially in CCA patients that are lacking the well-known tumor marker CA-19/9. Therefore, investigations of CA125 serum levels in clinical routine diagnostics are warranted as an additional and readily accessible biological resource.

In pancreatic cancer, combined chemo-immunotherapy with anti-MUC16 led to the development of specific T-cell immunity [19] while targeting MUC16 revealed prolonged progression-free and OS in a Phase II study in advanced ovarian cancer [30]. Next, the overcoming chemoresistance by targeting MUC16 may be beneficial in lung cancer, since MUC16 overexpression influences gemcitabine and cisplatin resistance in this entity [31]. Future studies should determine a specific therapeutic response for MUC16 (+) patients to enable the development of chemopreventive strategies in CCA. Interestingly, a correlation between therapeutical response and the quantity of MUC16 in ovarian cancer patients could be observed in two clinical trials [19,30]. In line with this, Wang et al. could show that patients with epithelial ovarian cancer and high MUC16 tissue expression had a significantly poorer prognosis compared to low MUC16-expressing patients [32]. Next, Gubbels et al. suggested that O-glycolisation on cell surface-bound MUC16 contributes to immune evasion in ovarian cancer cells. Interestingly, this observation depends on the MUC16 quantity since the expression of low levels of MUC16 correlated with an increased number of conjugates between malignant cells and natural killer cells [33]. Hence, the quantitative expression of MUC16 needs to be assessed and defined in further studies, as it could influence clinical outcome in CCA as well.

By MUC16 discrimination of iCCA cases, cell cycle and immune response processes are overrepresented among deregulated candidate transcripts. Enhanced cancer cell proliferation highly intersects with chemotherapy resistance processes. MUC16 (+) patients had higher Pn status and increased Ki-67 proliferative index underlining the functional annotation of our expression data. By predicting the activation of STAT3, we corroborate the frequently discussed association of MUC16 and JAK-STAT signaling [6,13,34]. This link might be orchestrated by Type I interferons (IFN) and mediate further transcription factors such as IFN regulatory factors (IRF) that also emerged in our expression analysis [35]. In line with this, PD-L1 expression increased in several cancer entities by IFN via JAK-STAT signaling [36,37]. Interestingly, recently published data reported increased survival rates for palliative CCA receiving standard of care chemotherapy if a PD-L1 inhibitor is added [38] while we confirmed clinically relevant CD274 (PD-L1) deregulation in MUC16 (+) patients by companion IHC diagnostics of PD-L1. Since data about the interplay between MUC16 and JAK-STAT signaling in CCA are missing so far, our preliminary gene expression profiling should, therefore, encourage further research investigating the underlying molecular mechanisms that are potentially altered in MUC16-expressing CCA.

We acknowledge the following limitations of our study. As a retrospective single center study, the sample size is modest and may lead to case selection bias, especially for pCCA and dCCA. Nevertheless, the case number of iCCA in our cohort is remarkably larger compared to Higashi et al. [16] while the number of cases for pCCA and dCCA is similar to Takihata et al. [17] and is limited due to the relatively rare occurrence of CCA in general. Since we analyzed data from a large TMA cohort, it should be considered that TMA cores only represent limited areas of tumor tissue. As we provide preliminary insights into genes that are particularly deregulated between MUC16-positive and -negative patients, we must acknowledge that this is a very small case series that is limited to iCCA only. Since the MUC16 antibody is well established in routine clinical practice for ovarian cancer, its applicability in CCA diagnosis is ensured without additional effort.

## 5. Conclusions

In summary, this study strengthens the limited published data on MUC16 expression in CCA tissue and its impact on survival by using a large cohort encompassing all CCA subtypes. MUC16 (+) is associated with impaired OS after surgical resection in curative intention, serving as an independent risk factor for poor prognosis. These findings strengthen MUC16 expression as a prognostically relevant marker for patients that are suffering from CCA and suggest including it in routine immunohistochemical staining.

## Figures and Tables

**Table 1:** Baseline characteristics of MUC16 (+)/(−) CCA patients.

Characteristics	MUC16 (−) (n = 111) No. (%)	MUC16 (+) (n = 57) No. (%)	*p*-Value
Sex			0.647
Female	39 (35.1)	18 (31.6)	
Male	72 (64.9)	39 (68.4)	
Age at initial diagnosis			0.075
Mean, years, (range)	64.3 (38–86)	67.3 (41–84)	
CCA subtype			<0.001
iCCA	80 (72.1)	23 (40.4)	
pCCA	19 (17.1)	16 (28.1)	
dCCA	12 (10.8)	18 (31.6)	
ECOG			0.621
0	76 (68.5)	37 (64.9)	
1	32 (28.8)	31 (31.6)	
2	3 (2.7)	2 (3.5)	
CA-19/9 (ng/mL)			0.011
<37	47 (42.3)	14 (24.6)	
≥37	41 (36.9)	32 (56.1)	
n.a.	23 (20.7)	11 (19.3)	
CA125 (U/mL)			0.301
<35	4 (3.6)	1 (1.8)	
≥35	0 (0)	3 (5.3)	
n.a.	107 (96.4)	53 (93)	
Tumor size (cm)			<0.001
≤5	52 (46.8)	46 (80.7)	
>5	59 (53.2)	11 (19.3)	
Single Tumor			0.567
Yes	73 (65.8)	40 (70.2)	
No	38 (34.2)	17 (29.8)	
Pathological grade			0.707
Grade 1	1 (0.9)	2 (3.5)	
Grade 2	83 (74.8)	38 (66.7)	
Grade 3	27 (24.3)	17 (29.8)	
M status			0.788
M0	104 (93.7)	54 (94.7)	
M1	7 (6.3)	3 (5.3)	
R status			0.257
R0	87 (78.4)	40 (70.2)	
R1	21 (18.9)	15 (26.3)	
Rx	3 (2.7)	2 (3.5)	
L status			0.439
L0	52 (46.8)	26 (45.6)	
L1	38 (34.2)	25 (43.9)	
Lx	21 (18.9)	6 (10.5)	
Pn status			0.034
Pn0	36 (32.4)	12 (21.1)	
Pn1	51 (45.9)	39 (68.4)	
Pnx	24 (21.6)	6 (10.5)	
Recurrence			0.766
Yes	46 (41.4)	25 (43.9)	
No	65 (58.6)	32 (56.1)	
Cholelithiasis			0.788
Yes	7 (6.3)	3 (5.3)	
No	104 (93.7)	54 (94.7)	
PSC			0.086
Yes	2 (1.8)	4 (7)	
No	109 (98.2)	53 (93)	
Viral hepatitis			0.884
Yes	9 (8.1)	5 (8.8)	
No	102 (91.9)	52 (91.2)	
Diabetes			0.464
Yes	27 (24.3)	11 (19.3)	
No	84 (75.7)	46 (80.7)	
Liver cirrhosis			0.053
Yes	7 (6.3)	0 (0)	
No	104 (93.7)	57 (100)	
Disease survival			0.385
Yes	45 (40.5)	12 (21.2)	
No	29 (26.1)	26 (45.6)	
Lost to follow-up	17 (15.3)	6 (10.5)	
n.a.	20 (18)	13 (22.8)	
Ki-67 (%)			
Mean, (range)	7.7 (0–40)	11.9 (0–80)	0.027

Positive M1 status reflects an intraoperative finding of M1 situation (e.g., distant lymph node metastasis) that was not known before surgery. Abbreviations: carbohydrate antigen 19-9 (CA-19/9), Eastern Cooperative Oncology Group (ECOG), intrahepatic cholangiocarcinoma (iCCA), distal cholangiocarcinoma (dCCA), not available (n.a.), number (No.), perihilar cholangiocarcinoma (pCCA), primary sclerosing cholangitis (PSC).

**Table 2:** Univariate and multivariate Cox regression analysis of OS in patients with CCA.

	Univariate Analysis			Multivariate Analysis		
Characteristics	HR	95% CI	*p*-Value	HR	95% CI	*p*-Value
Sex						
Female	ref					
Male	1.293	0.889–1.88	0.179			
CCA subtype						
iCCA	ref					
pCCA	1.18	0.769–1.812	0.449			
dCCA	1.162	0.749–1.802	0.503			
ECOG						
0	ref			ref		
1	2.674	1.813–3.944	<0.001	2.031	1.265–3.262	0.003
2	1.728	0.544–5.488	0.354	1.148	0.35–3.769	0.82
CA-19/9 (ng/mL)						
<37	ref			ref		
≥37	2.136	1.422–3.208	<0.001	1.646	1.036–2.615	0.035
Tumor size (cm)						
≤5	ref					
>5	1.021	0.717–1.456	0.907			
Single Tumor						
Yes	ref			ref		
No	1.795	1.242–2.594	0.002	1.714	1.069–2.747	0.025
MUC16						
Negative	ref			ref		
Positive	1.937	1.337–2.806	<0.001	1.636	1.043–2.568	0.032
Pathological grade						
Grade 1	ref			ref		
Grade 2	1.521	0.374–6.194	0.558	0.672	0.086–5.272	0.705
Grade 3	4.806	1.152–20.052	0.031	1.779	0.219–14.48	0.59
M status						
M0	ref			ref		
M1	2.688	1.466–4.928	0.001	1.801	0.836–3.88	0.133
R status						
R0	ref			ref		
R1	1.608	1.087–2.379	0.018	1.06	0.637–1.764	0.822
Recurrence						
No	ref					
Yes	1.16	0.818–1.646	0.405			
PSC						
No	ref					
Yes	1.734	0.762–3.946	0.19			
Diabetes						
No	ref					
Yes	1.141	0.759–1.716	0.525			
Viral hepatitis						
No	ref					
Yes	0.541	0.252–1.161	0.115			
Liver cirrhosis						
No	ref					
Yes	0.744	0.274–2.02	0.562			

Positive M1 status reflects an intraoperative finding of the M1 situation (e.g., distant lymph node metastasis) that was not known before surgery. Abbreviations: carbohydrate antigen 19-9 (CA-19/9), cholangiocarcinoma (CCA), confidence interval (CI), Eastern Cooperative Oncology Group (ECOG), hazard ratio (HR), intrahepatic cholangiocarcinoma (iCCA), distal cholangiocarcinoma (dCCA), perihilar cholangiocarcinoma (pCCA), primary sclerosing cholangitis (PSC).

## Data Availability

The data are available upon reasonable request from the corresponding author.

## Supplementary Materials

The following supporting information can be downloaded at: https://www.mdpi.com/article/10.3390/cancers14194703/s1, Figure S1: Prominently deregulated transcripts of MUC16 (+) vs. baseline MUC16 (−) iCCA; Figure S2: Network prediction analysis using Ingenuity pathway analysis by merging networks that are associated with cancer growth and survival; Figure S3: Representative control image of MUC16 expression in cancerous and paracancerous CCA tissue; Table S1: The most differentially expressed genes among MUC16-positive and -negative iCCA; Table S2: Top genes from all clusters that are associated with the five representative terms and pathways using REACTOME_pathways ontology database; Table S3: Additional differentially expressed genes among MUC16-positive and -negative iCCA; Table S4: Detailed overview of different patterns of recurrence.
