# Supplementary material for: Expression of MUC16/CA125 Is Associated with Impaired Survival in Patients with Surgically Resected Cholangiocarcinoma
Source: Cancers (Basel). 2022 Sep 27;14(19):4703. doi: 10.3390/cancers14194703 (PMC9563928; doi:10.3390/cancers14194703)
Supplement: Supplementary file 1 [file cancers-14-04703-s001.zip › cancers-1913884-supplementary.pdf]

Supplementary Figures and Tables

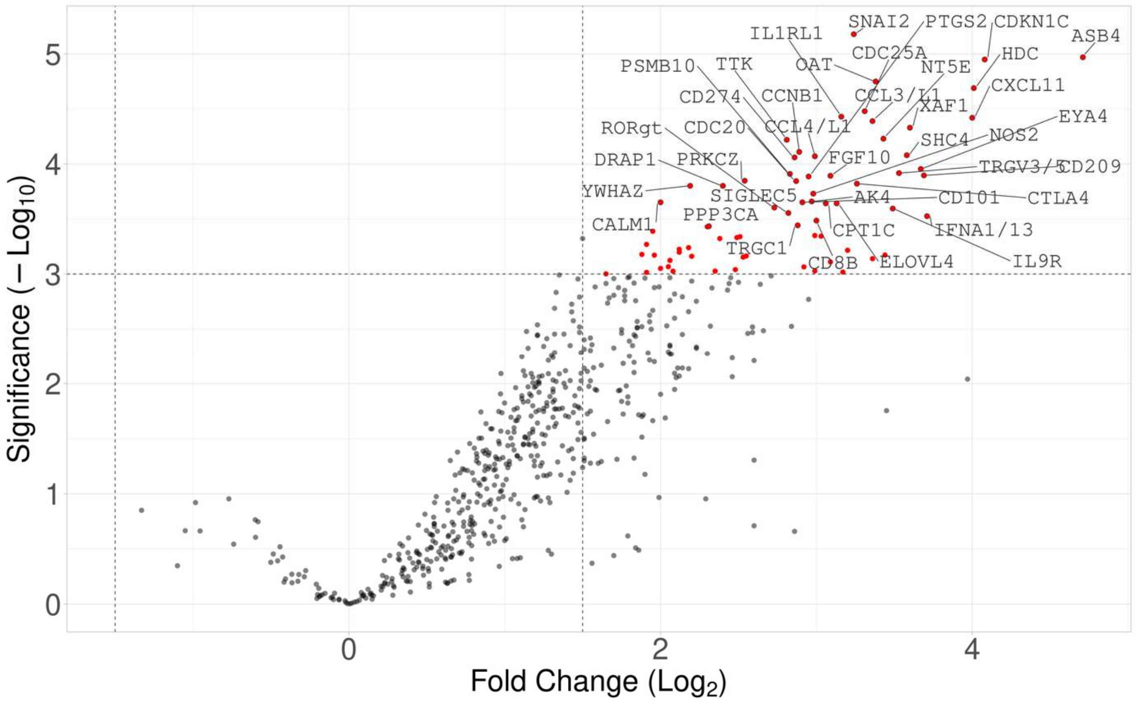

Figure S1. Prominently deregulated transcripts of MUC16 (+) vs. baseline MUC16 (-) iCCA. Volcano plot displaying each gene's -log10(p-Value) and log2 fold change with the selected covariate. Highly statistically significant genes fall at the top of the plot above the horizontal lines, and highly differentially expressed genes fall to either side. Horizontal lines indicate various FDR thresholds or p-Value thresholds if there is no adjustment to the p-Values. Genes are colored if the resulting p-Value is below the given FDR or p-Value threshold. The 40 most statistically significant genes are labeled in the plot.

Abbreviations: intrahepatic Cholangiocarcinoma (iCCA), False Discovery Rate (FDR)

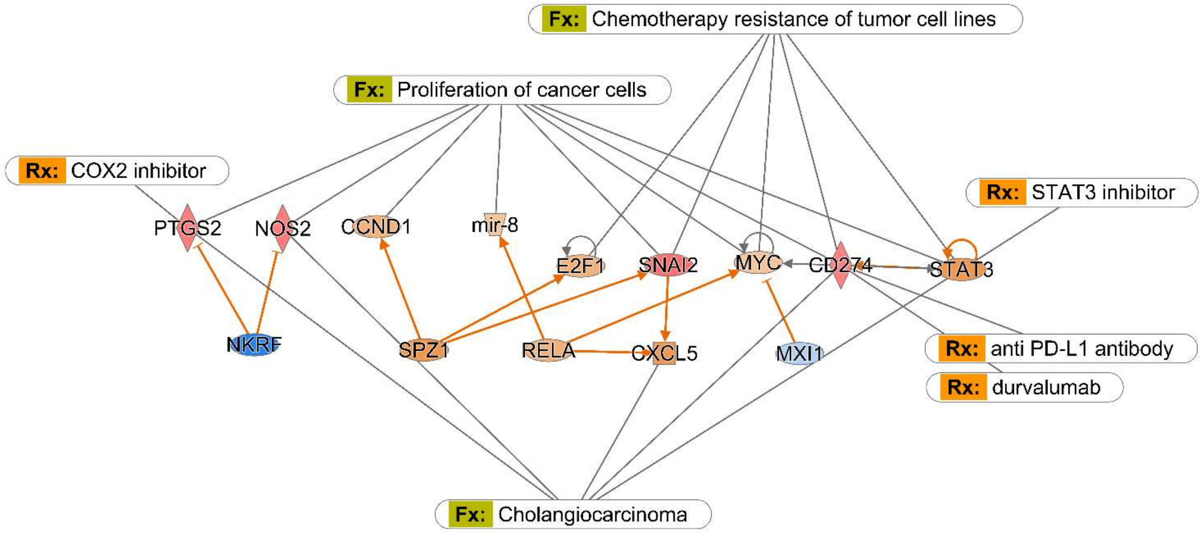

Figure S2. Network prediction analysis using Ingenuity pathway analysis by merging networks associated with cancer growth and survival. PTGS2, NOS2, SNAI2 and CD274 (PD-L1) predict further biomarkers associated with proliferation of cancer cells highly intersecting with chemotherapy resistance processes. Among predicted molecules, some have been reported in association with CCA and available therapeutic agents.
Red node color, elevated log2 fold changes; orange node color, predicted activation; blue node color, predicted inhibition; orange relation predicts activation.

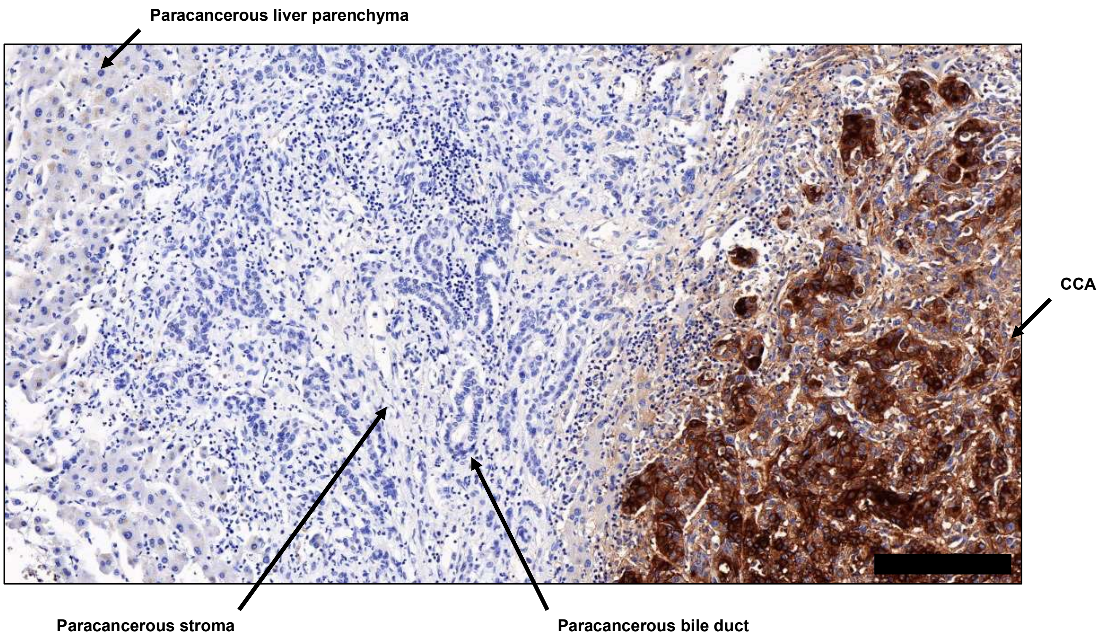

Figure S3. Representative control image of MUC16 expression in cancerous and paracancerous CCA tissue. Immunohistochemistry of MUC16 in MUC16 (+) CCA and paracancerous liver parenchyma, normal bile duct and stroma tissue. Original magnification x12.5, scale bar: 200 µm.

Abbreviations: Cholangiocarcinoma (CCA)

Table S1. The most differentially expressed genes among MUC16 positive and negative iCCA. 'Estimated log fold-change' estimates a gene's differential expression. For categorical covariates, a gene is estimated to have 2^(log fold change) times its expression in MUC16 (-) baseline samples, holding all other variables in the analysis constant. The log2 and linear fold change is also presented, along with a p-Value and an adjusted p-Value or FDR (BY correction).

Gene Symbol	Log2 fold change	*p*-Value	BY *p*-Value	Gene Sets
ASB4	4.71	1.06E-05	0.0167	Antigen Presentation
CDKN1C	4.08	1.13E-05	0.0167	Cell Cycle
HDC	4.01	2.02E-05	0.0178	n.a.
CXCL11	4	3.81E-05	0.0198	Chemokine Signaling, TLR Signaling
IFNA1/13	3.71	0.000298	0.0355	Cytotoxicity, JAKSTAT Signaling, PI3K-AKT Pathway, TLR Signaling
CD209	3.69	0.000127	0.0259	Myeloid Immune Evasion
EYA4	3.67	0.000111	0.0259	Epigenetic Modification
XAF1	3.6	4.71E-05	0.0208	Type I Interferon
SHC4	3.58	8.28E-05	0.024	Cytotoxicity
TRGV3/5	3.53	0.000121	0.0259	TCR Signaling
IL9R	3.49	0.000254	0.032	IL-2 Signaling
NT5E	3.43	5.92E-05	0.0221	Myeloid Immune Evasion
OAT	3.38	1.78E-05	0.0178	Glutamine Metabolism
CCL3	3.36	4.03E-05	0.0198	Chemokine Signaling, IL-10 Signaling, T Cell Exhaustion, TLR Signaling
CDC25A	3.31	3.30E-05	0.0198	Cell Cycle, Senescence & Quiescence
CTLA4	3.26	0.000151	0.0259	CTLA4 Signaling, NK Exhaustion, T Cell Checkpoint Signaling, T Cell Exhaustion, TCR Signaling
SNAI2	3.24	6.60E-06	0.0167	Epigenetic Modification
IL1RL1	3.16	3.73E-05	0.0198	IL-1 Signaling
ELOVL4	3.13	0.000228	0.0306	Fatty Acid Metabolism
FGF10	3.09	0.000128	0.0259	MAPK Signaling
CPT1C	3.06	0.000228	0.0306	Fatty Acid Metabolism, PPAR signaling
CD8B	3	0.000327	0.038	TCR Signaling
CCL4	2.99	8.43E-05	0.024	Chemokine Signaling, IL-10 Signaling, NF-kB Signaling, TLR Signaling
NOS2	2.98	0.000186	0.0293	Hypoxia Response, Myeloid Immune Evasion, Other Interleukin Signaling
AK4	2.97	0.000219	0.0306	Hypoxia Response
PTGS2	2.95	0.00013	0.0259	Hypoxia Response, IL-10 Signaling, Myeloid Immune Evasion, NF-kB Signaling, Other Interleukin Signaling, TNF Signaling
CD101	2.91	0.000223	0.0306	TCR Signaling
CCNB1	2.89	7.82E-05	0.024	Cell Cycle, Epigenetic Modification, Senescence & Quiescence
TRGC1	2.88	0.000361	0.04	TCR Signaling
CDC20	2.87	0.000143	0.0259	Cell Cycle

Gene Symbol	Log2 fold change	*p*-Value	BY *p*-Value	Gene Sets
CD274	2.86	8.70E-05	0.024	PD1 Signaling, T Cell Checkpoint Signaling
PSMB10	2.83	0.000123	0.0259	Antigen Presentation, BCR Signaling
TTK	2.81	6.01E-05	0.0221	Cell Cycle
SIGLEC5	2.82	0.000279	0.0342	n.a.
ROR	2.73	0.000249	0.032	Other Interleukin Signaling
PRKCZ	2.54	0.000142	0.0259	TGF-beta Signaling
DRAP1	2.4	0.000158	0.0259	TGF-beta Signaling
PPP3CA	2.31	0.000368	0.04	BCR Signaling, MAPK Signaling, Senescence & Quiescence, TCR Signaling
YWHAZ	2.19	0.000158	0.0259	Cell Cycle, Notch Signaling, Other Interleukin Signaling, PI3K-AKT Pathway
CALM1	2	0.000223	0.0306	BCR Signaling, Senescence & Quiescence

Abbreviations: Benjamini-Yekuteli (BY), False Discovery Rate (FDR), intrahepatic Cholangiocarcinoma (iCCA), not available (n.a.)

Table S2. Top Genes from all Clusters associated to 5 representative terms and pathways using REACTOME_pathways ontology database. 33 of top 40 prominently differentially regulated genes are statistically overrepresented in a functional network.

GOID	GOTerm	% Associated Genes	Nr. Genes	Associated Genes Found
R-HSA:1280218	Adaptive Immune System	1.55	12.00	[ASB4, CALM1, CD101, CD209, CD274, CD8B, CDC20, CTLA4, PPP3CA, PSMB10, SIGLEC5, YWHAZ]
R-HSA:168256	Immune System	1.03	21.00	[ASB4, CALM1, CCL3, CCL4, CD101, CD209, CD274, CD8B, CDC20, CTLA4, IFNA1, IFNA13, IL1RL1, IL9R, NOS2, PPP3CA, PSMB10, PTGS2, SIGLEC5, XAF1, YWHAZ]
R-HSA:69620	Cell Cycle Checkpoints	1.71	5.00	[CCNB1, CDC20, CDC25A, PSMB10, YWHAZ]
R-HSA:9006925	Intracellular signaling by second messengers	1.61	5.00	[CALM1, FGF10, IL1RL1, PSMB10, SNAI2]
R-HSA:1280215	Cytokine Signaling in Immune system	1.51	11.00	[CCL3, CCL4, IFNA1, IFNA13, IL1RL1, IL9R, NOS2, PSMB10, PTGS2, XAF1, YWHAZ]
R-HSA:449147	Signaling by Interleukins	1.68	8.00	[CCL3, CCL4, IL1RL1, IL9R, NOS2, PSMB10, PTGS2, YWHAZ]

Table S3. Additional differentially expressed genes among MUC16 positive and negative iCCA. Shown are selected targets which are amongst predicted biomarkers and downstream processes.

Gene Symbol	Log2 fold change	*p*-Value	BY *p*-Value	Gene Sets
IRF4	2.56	0.00347	0.114	Epigenetic Modification, Other Interleukin Signaling, T Cell Exhaustion, Type I Interferon, Type II Interferon
IRF2	1.51	0.00726	0.178	Type I Interferon, Type II Interferon
IRF8	0.525	0.305	1	Type I Interferon, Type II Interferon
STAT3	1.15	0.00474	0.135	Hypoxia Response, JAKSTAT Signaling
STAT5A	1.25	0.0168	0.297	JAKSTAT Signaling
STAT1	0.989	0.106	1	JAKSTAT Signaling, Notch Signaling, TLR Signaling
STAT5B	0.383	0.251	1	JAKSTAT Signaling, NK Activity, NK Exhaustion
STAT4	0.618	0.382	1	JAKSTAT Signaling
JAK3	1.01	0.074	0.842	JAKSTAT Signaling
JAK2	0.842	0.0835	0.936	Epigenetic Modification, IL-6 Signaling, JAKSTAT Signaling, Type II Interferon
JAK1	0.634	0.121	1	JAKSTAT Signaling
RELA	1.48	0.00303	0.108	NF-kB Signaling
E2F5	-0.485	0.351	1	Cell Cycle, Senescence & Quiescence, TGF-beta Signaling
E2F2	0.475	0.453	1	Cell Cycle, Senescence & Quiescence
E2F1	-0.0625	0.905	1	Cell Cycle, Hypoxia Response, Notch Signaling, Senescence & Quiescence

Abbreviations: Benjamini-Yekuteli (BY), intrahepatic Cholangiocarcinoma (iCCA)

Table S4. **Detailed overview of different patterns of recurrence.**

Patterns of recurrence	MUC16 (-) (n=46) No. (%)	MUC16 (+) (n=25) No. (%)
Lymph node	1 (2.2)	2 (8)
Liver	26 (56.5)	13 (52)
Pulmonary	3 (6.5)	3 (12)
Bone	3 (6.5)	0 (0)
Peritoneal	1 (2.2)	2 (8)
Liver + bone	2 (4.3)	0 (0)
Liver + pulmonary	2 (4.3)	2 (8)
Liver + peritoneal	0 (0)	2 (8)
Liver + lymph node	3 (6.5)	0 (0)
Liver + lymph node + bone	3 (6.5)	0 (0)
Liver + lymph node + peritoneal	1 (2.2)	0 (0)
Liver + pulmonary + peritoneal	0 (0)	1 (4)
Pulmonary + bone + peritoneal	1 (2.2)	0 (0)

Abbreviations: No. (Number)
